# Correction: Evolutionary principles of modular gene regulation in yeasts

**DOI:** 10.7554/eLife.01114

**Published:** 2013-07-02

**Authors:** Dawn A Thompson, Sushmita Roy, Michelle Chan, Mark P Styczynski, Jenna Pfiffner, Courtney French, Amanda Socha, Anne Thielke, Sara Napolitano, Paul Muller, Manolis Kellis, Jay H Konieczka, Ilan Wapinski, Aviv Regev

Thompson DA, Roy S, Chan M, Styczynski MP, Pfiffner J, French C, Socha A, Thielke A, Napolitano S, Muller P, Kellis M, Konieczka JH, Wapinski I, Regev A. 2013. Evolutionary principles of modular gene regulation in yeasts. *eLife* **2**:e00603. doi: http://dx.doi.org/10.7554/eLife.00603. Published 18 June 2013

Mark P Styczynski’s name was incorrectly spelled ‘Styczynsky’.

The second sentence of the abstract incorrectly gave the time course of growth of *Ascomycota* as 1 billion years. This has been corrected to 300 million years.

The article has been corrected accordingly.

